# D-Mannose Suppresses γδ T Cells and Alleviates Murine Psoriasis

**DOI:** 10.3389/fimmu.2022.840755

**Published:** 2022-02-28

**Authors:** Mingyang Li, Haiyan Cheng, Dan Tian, Lu Yang, Xiaonan Du, Yuhualei Pan, Dong Zhang, Xueling Mei

**Affiliations:** ^1^ Immunology Research Center for Oral and Systemic Health, Beijing Friendship Hospital, Capital Medical University, Beijing, China; ^2^ Beijing Key Laboratory of Tolerance Induction and Organ Protection in Transplantation Beijing Friendship Hospital, Capital Medical University, Beijing, China; ^3^ Immunology Research Center, Beijing Clinical Research Institute, Beijing, China; ^4^ General Surgery Department, Beijing Friendship Hospital, Capital Medical University, Beijing, China; ^5^ Department of Dermatology, Beijing Friendship Hospital, Capital Medical University, Beijing, China

**Keywords:** psoriasis, γδ T cells, D-mannose, IL-17, glycolysis

## Abstract

Psoriasis is a chronic skin disorder associated with multiple sequelae, such as psoriatic arthritis and cardiovascular diseases. Increasing evidence has shown that γδ T cells, as sources of IL-17A, play critical roles in psoriatic inflammations. However, there still lack effective ways to manipulate these pathogenic γδ T cells, which are less well studied than αβ T cells. The present study aims to characterize the phenotype of γδ T cells and evaluate the impact of D-mannose (a C-2 epimer of glucose) on γδ T cell-mediated psoriasis. We found that skin-draining LN γδ T cells underwent robust proliferation and acquired an IL-17-producing phenotype during psoriasis. The transcriptomic profiles of these psoriatic γδ T cells had elevated glycolytic signatures. Importantly, D-mannose treatment suppressed the γδ T cell reaction and successfully alleviated the local and systematic inflammation induced by imiquimod. The decreased AKT/mTOR/HIF-1α signaling and glycolytic ability may contribute to the suppression of γδ T cells achieved by D-mannose. Our study increased understanding of γδ T cells in psoriasis and promoted D-mannose utilization as a potential clinical application for autoimmune diseases driven by γδ T cells.

## Introduction

Psoriasis is a persistent immune-mediated inflammatory skin disorder affecting 2-3% of the population worldwide (1–3). In addition to the common characteristics of squamous reddish plaques on extensor surfaces, psoriasis is also associated with sequelae such as psoriatic arthritis, cardiometabolic disorders and psychiatric diseases (1, 2, 4, 5). The pathologies of psoriasis are intricate and not fully explicit (6). However, excessive activation of innate and adaptive immune reactions is centrally involved (3, 6, 7). The multidimensional interactions between innate immune cells (dendritic cells, neutrophils), adaptive immune cells and skin resident cells (keratinocytes) through cell contact and cytokines (IL-17, etc.) aggravate the psoriatic injuries (8–11). Among the adaptive effector cells, T cells play an essential role in the pathology of skin disorders by activating keratinocytes through the IL-23/IL-17 axis (11, 12). Conventionally, TCRαβ^+^ Th cells have received plentiful attentions, but increasing evidences have shown that IL-17-secreting TCRγδ^+^ (γδ17) T cells are pivotal mediators in psoriasis (13–15). In both IL-23-induced and imiquimod (IMQ)-induced models, γδ T cell deletion rather than αβ T cell deletion alleviated skin inflammations (16). In addition, the primary sources of the pathogenic IL-17 superfamily in psoriatic lesions were found to be γδ T cells instead of αβ T cells (16, 17). Thus, these evidences proved the significant importance of γδ T cells in psoriatic occurrence and progression. After IMQ administration, TCR Vγ4^+^ γδ17 T cells may rapidly expand in skin draining lymph node (DLN) and egress out to exacerbate skin inflammation (18, 19). Compared to TCR Vγ6^+^ dermal T cells, which also produce IL-17, the Vγ4^+^ T cells released from the DLN have significantly greater capabilities to proliferate and produce IL-17 (18, 19). Hence, γδ17 T cells from the skin DLN were thought to have a critical role in IMQ-induced psoriasis.

Upon antigen stimulation, T cells undergo robust clonal expansion and differentiation (20). Their fates are usually decided by transcriptional and epigenetic modifications (21, 22). Under conditions favoring Th17 differentiation, hypoxia-inducible factor-1α (HIF-1α) is usually upregulated to activate RORγt and promote the production of IL-17 (23, 24). Meanwhile, as a pluripotent transcription factor, HIF-1α is also implicated in the process of glycolysis, which supports the huge bioenergetic demands of Th17 cells (23, 25). Suppressing HIF-1α-dependent glycolysis could impede the development of Th17 cells and delay the progression of experimental autoimmune encephalomyelitis (25, 26); therefore, we speculate that manipulation of HIF-1α and glycolysis may also be a potent way to relieve the psoriasis induced by γδ17 T cells. D-mannose, a C-2 epimer of glucose, has been used as a nonantibiotic treatment for urinary infection by blocking the adhesion of bacteria to epithelial cells (27, 28). Recent works have shown that D-mannose can impair glycolysis and promote fatty acid oxidation in T cells (29). In turn, it can induce regulatory T cells (Tregs) and alleviate the immunopathology of diabetes, airway inflammation and lupus (28–30). More importantly, the suppression of succinate-mediated HIF-1α activation was reported in macrophages treated with D-mannose (31). These data supported the clinical application of D-mannose in autoimmune diseases. However, whether D-mannose treatment can suppress psoriasis induced by γδ T cells remains to be determined.

In this study, we compared the phenotypes, functions, and transcriptional alterations of γδ T cells in IMQ-induced psoriasis. In addition to intensive activation and proliferation, we found that γδ T cells from the skin DLN of psoriatic mice had a higher level of p-HIF-1α expression and upregulated genes associated with glycolysis. Moreover, D-mannose treatment successfully attenuated psoriatic inflammation. Through our findings, we speculated that the decreased AKT/mTOR/HIF-1α signaling and glycolytic ability in γδ T cells may contribute to the suppression of psoriasis achieved by D-mannose.

## Materials and Methods

### Animal Models

Eight-week-old male C57BL/6 mice (weighing 20g-25g) were purchased from HFK Laboratory (Beijing, China). After being allowed to adapt for 5 days, the mice were randomized assigned to different groups using the random number table. The numbers of mice used in each group were shown in figure legends. For the establishment of psoriasis model, mice were shaved and treated with a topical dose of 62.5 mg of imiquimod (IMQ) cream (3 M, USA) or vehicle cream (Ctrl) on their back for six consecutive days. At Day 7, mice were sacrificed. Their skin was fixed in 4% paraformaldehyde and then embedded in paraffin for HE staining. To evaluate the impact of D-mannose on IMQ-induced psoriasis, mice were administered 200 μl of 20% (w/v) D-mannose (1.1M) (Sigma, USA) by gavage twice a day while given drinking water with 20% D-mannose (1.1M) ad libitum for one week. Then, the IMQ-induced model was established, while D-mannose was still administered orally. After 6 consecutive days of IMQ treatment, the mice were harvested. The dosage of D-mannose used in our research was previously reported to have no adverse effect on the weight and health of the animals (29–32). All mice were housed in a pathogen-free, comfortable temperature environment with a 12 h light/dark cycle. All animal studies were performed in compliance with the ethical guidelines for animal studies and approved by the Animal Ethics Committee of Beijing Friendship Hospital, Capital Medical University (approval no. 20-2018).

### Psoriasis Area and Severity Index (PASI) Assessment

The PASI score was used to assess the severity of the lesion upon IMQ exposure. The score was graded from 0 to 4 according to the erythema, scale and thickness of the skin. A score of 0 means normal, and a score of 4 means very severely altered. Every mouse was graded according to PASI daily, and the assessment was performed blinded.

### Reagents

Antibodies against TCRγδ (GL3), TCRβ (H57-597), CD3 (17A2), Foxp3 (MF23), CD25 (PC61), PD1 (J43), Annexin V, IFN-γ (XMG1.2), NK1.1 (S17016D), CD27 (LG.3A10), IL-23R (12B2B64), IL-17A (TC11-18H10), CD45 (A20), CCR2 (SA203G11), CCR5 (HM-CCR5), CCR6 (29-2L17), CXCR6 (SA051D1), ICOS (C398.4A), CD62L (MEL-14), Ki-67 (SoIA15), CD44 (IM7), IRF4 (IRF4.3E4), and Blimp1 (5E7) were purchased from Biolegend. Antibodies against p-AKT (Ser473) (SDRNR), p-HIF-1α (Mgc3), and p-mTOR (Ser2448) (MRRBY) were purchased from Thermo Fisher. Before cell staining, TruStain FcX™ PLUS (anti-mouse CD16/32) antibodies (Biolegend) were used to block nonspecific binding. For intracellular staining of cytokines, the cells were stimulated with Cell Activation Cocktail (with Brefeldin A) from Biolegend. Six hours later, the cells were stained with antibodies against surface molecules and prepared according to the instructions of the Cyto-Fast™ Fix/Perm Buffer Set (Biolegend). For intracellular staining of transcription factors and signaling molecules, the cells were prepared with a Foxp3/Transcription Factor Buffer Set (Thermal Fisher).

### Cell Culture

γδ T cells sorted from the spleens and peripheral LNs of 15 healthy mice were pooled together and cultured (2×10^5^ per well) for 72 hours with IL-2 (2 ng/ml) in a round-bottom 96-well plate. Antibodies against CD3 (10 μg/ml) and CD28 (1 μg/ml) were added. To evaluate the impact of D-mannose on γδ T cells, cells were also treated with D-mannose (50 mmol/L).

### Glycolytic Rate Assay

To determine the impact of D-mannose on γδ T cell glycolytic capabilities, γδ T cells were pretreated with or without D-mannose *in vitro* as described above for three days. Subsequently, these cells were harvested and seeded on a poly-D-lysine-coated 96-well XF microplate (2-4×10^5^ cells/well) and cultured in XF RPMI 1640 medium with glucose (25 mmol/L), pyruvate (1 mmol/L) and glutamine (4 mmol/L). In the D-mannose assay group, D-mannose (25 mmol/L) was added to the culture medium. Finally, to measure the extracellular acidification rate (ECAR) of these γδ T cells, an Agilent Seahorse XFe-96 metabolic analyzer and glycolytic rate assay kit (Agilent) were used according to the instructions. The level of ECAR may reflect the capabilities of glycolysis in γδ T cells.

### Transcriptome Analysis

RNA samples from skin-draining LN and splenic γδ T cells (from healthy and psoriatic mice) were sequenced using a standard Illumina protocol (Annoroad Gene Technology, Beijing). Each sample represented γδ T cells obtained from 15 mice. Reads were mapped to a mouse genome (Mm9) by using HISAT2. Gene counts were estimated by HTSeq. The R package DESeq2 was applied to determine differentially expressed genes (DEGs). Genes with a fold-change >2 and an adjusted *P* value < 0.05 were defined as DEGs. The R package clusterprofiler was applied to perform GO enrichment and GSEA of the DEGs. The data reported in this work have been uploaded to the Gene Expression Omnibus (GEO) database under accession number GSE188905.

### RT–PCR

RNA was extracted from γδ T cells using the RNeasy Plus Micro Kit (Qiagen), and cDNA was obtained using PrimeScript™ RT Master Mix (TaKaRa). Quantitative PCR was performed using Hieff qPCR SYBR Green Master mix (YEASEN, Beijing) on a QuantStudio™ 3 Real-Time PCR Instrument (Thermal Fisher), with each sample in triplicate. The quantification was based on 2^-ΔΔCt^ calculations and was normalized to β-actin. The primers used in the article were listed in **Table S1**.

### Capillary Western Blot

The protein of γδ T cells cultured *in vitro* was extracted by RIPA lysis buffer (Roche). Each sample represented γδ T cells obtained from 5 mice. After quantified the concentration of protein by bicinchoninic acid assay (Thermal Fisher), the expressions of p-AKT (Ser473), p-mTOR (Ser2448), and β-acitn (8H10D10) were evaluated using a capillary western blot analyzer (ProteinSimple). The 25-lane plates were used according to the instructions. All the antibodies for western blot were purchased from Cell Signaling Technology.

### Statistical Analysis

GraphPad Prism 8 software was applied to perform the statistical analysis. An unpaired Student’s *t* test was used to evaluate the significance of the difference between two groups. For comparisons between multiple groups, the two-way ANOVA with Sidak’s multiple comparison test was performed. Data were presented as the mean ± SEM, and a *P* value < 0.05 was considered significant (*p < 0.05, **p < 0.002, ***p < 0.0002, ****p < 0.0001). The *t* values and *F* values of each comparison were also shown in the figure legends.

## Results

### γδ T Cells Were Significantly Expanded in IMQ-Treated Mice

**Figure 1:**
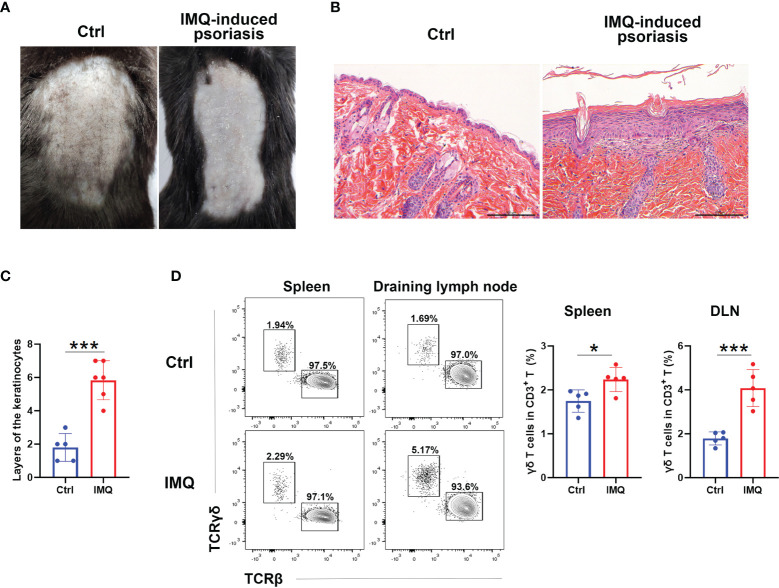
γδ T cells expanded in IMQ-treated mice. Mice were shaved and treated with imiquimod (IMQ) on their backs for six consecutive days. At Day 7, they were harvested. **(A)** Pictures of control (Ctrl) and IMQ-treated mice. **(B)** Representative H&E staining of skin sections obtained from the Ctrl- and IMQ-treated mice. The scale bar represents 100 μm. **(C)** Statistical analysis of the layers of keratinocytes in HE sections obtained from Ctrl- and IMQ-treated mice (N=5-6, *t*=6.438). **(D)** Flow cytometry analysis of TCRγδ^+^ cells and TCRβ^+^ cells in the spleen and skin-draining lymph node (DLN). The comparison of the TCRγδ^+^ cell proportions in spleen (*t*=2.949) and DLN (*t*=5.744) obtained from Ctrl- and IMQ-treated mice is shown on the right (N=5). The Student’s *t* test was used to compare the differences mentioned above. At least three independent experiments were performed with five mice in each group. *p < 0.05, ***p < 0.0002.

To investigate the alteration of γδ T cells, we first treated the mice with IMQ on their backs for six consecutive days. As shown in **Figures 1A–C**, IMQ treatment resulted in signs of scurf and thickened skin (**Figure 1A**). Histological analysis of skin sections showed that IMQ-treated mice had parakeratosis and thickened dermal and inflammatory cell infiltrations (**Figures 1B, C**). These results indicated that the psoriasis model was successfully established. We then compared the ratio of γδ T cells to CD3^+^ T cells in skin-draining LNs (DLNs) and spleens between IMQ-treated and control mice (**Figure 1D**). The gating strategy of γδ T cells for flow cytometry analysis was shown in **Figure S1**. Upon IMQ treatment, the frequency of γδ T cells was increased in both DLN and spleen; however, the frequency of γδ T cells in the DLN was higher, and the fold-change of the γδ T cell ratio in the DLN was more remarkable than that in the spleen (by 2.28 in the DLN and 1.30 in the spleen) (**Figure 1D**). These results were consistent with previous reports (16, 18) and showed that IMQ stimulation expanded γδ T cells in the DLNs and spleens.

### Skin-Draining LN γδ T Cells From IMQ-Treated Mice Had an Activated/Effector Phenotype Compared With Other γδ T Populations

**Figure 2:**
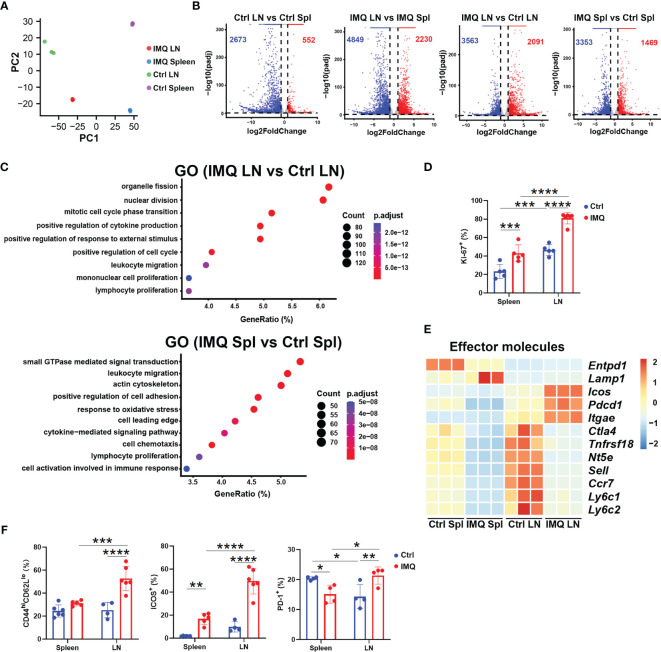
RNA-seq analysis of γδ T cells obtained from healthy and psoriatic mice. For sequencing, skin-draining LN (LN) and splenic (Spl) γδ T cells from healthy (Ctrl) and psoriatic (IMQ) mice were pooled separately. **(A)** PCA of γδ T cells from skin-draining LNs and spleens of healthy and psoriatic mice. Each sample represented γδ T cells obtained from 15 mice. **(B)** Volcano plot of γδ T cells from the groups described above. The numbers of upregulated and downregulated genes in each group are indicated as red and blue, respectively. **(C)** Pathways significantly enriched using highly expressed genes in the IMQ LN vs. Ctrl LN group and IMQ Spl vs. Ctrl Spl group by GO analysis. **(D)** Comparison of Ki-67^+^ cells in splenic and skin-draining LN γδ T cells was performed (N=5, *F*=5.446). **(E)** Heatmap of genes associated with effector functions. **(F)** Flow cytometry analysis of CD44^hi^CD62^lo^ (*F*=10.93), ICOS^+^ (*F*=17.09), and PD1^+^ (*F*=17.98) cells in γδ T cells from different tissues (N=4-6). The two-way ANOVA with Sidak’s multiple comparison was performed to compare the differences described above. *p < 0.05, **p < 0.002, ***p < 0.0002, ****p < 0.0001.

We next characterized the γδ T cells from the DLN and spleen by RNA-seq analysis. Both principal component analysis (PCA) and a dendrogram provided a clear demonstration that γδ T cells from different groups were distinct from each other (**Figure 2A** and **Figure S2A**). Compared to control mice, the γδ T cells from IMQ-treated mice had a total of 2091 upregulated genes and 3563 downregulated genes in the DLN, as well as 1469 and 3353, respectively, in the spleen (absolute fold change>2, *P.*adj<0.05) (**Figure 2B**). The transcriptional differences between DLN and splenic γδ T cells were also significantly amplified upon IMQ stimulation, from 552 upregulated genes and 2673 downregulated genes (Ctrl LN vs. Ctrl Spl) to 2230 upregulated genes and 4849 downregulated genes (IMQ LN vs. IMQ Spl) (**Figure 2B**). Comparing the differentially expressed genes (DEGs) from different groups by Gene ontology (GO) analysis, we found that the upregulated DEGs in the DLN were enriched in lymphocyte proliferation, leukocyte migration, positive regulation of the cell cycle and cytokine production; however, the upregulated DEGs in the spleen were primarily enriched in small GTPase-mediated signal transduction, leukocyte migration, and cell chemotaxis (**Figure 2C**). Further comparison of DEGs between DLN and splenic γδ T cells from IMQ-treated mice also showed that the γδ T cells in DLN attained a higher level of proliferation, while the splenic γδ T cells got enhanced in the capabilities such as cell chemotaxis and adhesion (**Figure S2B**). Consistent with the RNA-seq results, our flow cytometry analysis showed that the DLN γδ T cells from IMQ-treated mice had the highest level of Ki-67 expression among different γδ T populations, supporting the augmented proliferation of these cells (**Figure 2D** and **Figure S2C**).

As shown in **Figure 2E**, γδ T cells from IMQ DLNs demonstrated a higher level of activation/effector gene expression, including *Icos, Pdcd1, and Itgae*, and lower levels of *Sell* and *Ccr7* expression. Hence, we examined the cells by flow cytometry and found that the DLN γδ T cells from IMQ-treated mice included more activated T cells (CD44^hi^CD62L^lo^) with higher expression of ICOS and PD1 than other γδ T populations (**Figure 2F** and **Figure S2C**). Together, these results indicated that the inflammatory γδ T cells in the DLN of psoriatic mice are an activated population with highly proliferative features.

### Skin-Draining LN γδ T Cells From Psoriatic Mice Included More Cells With IL-17-Producing Features

**Figure 3:**
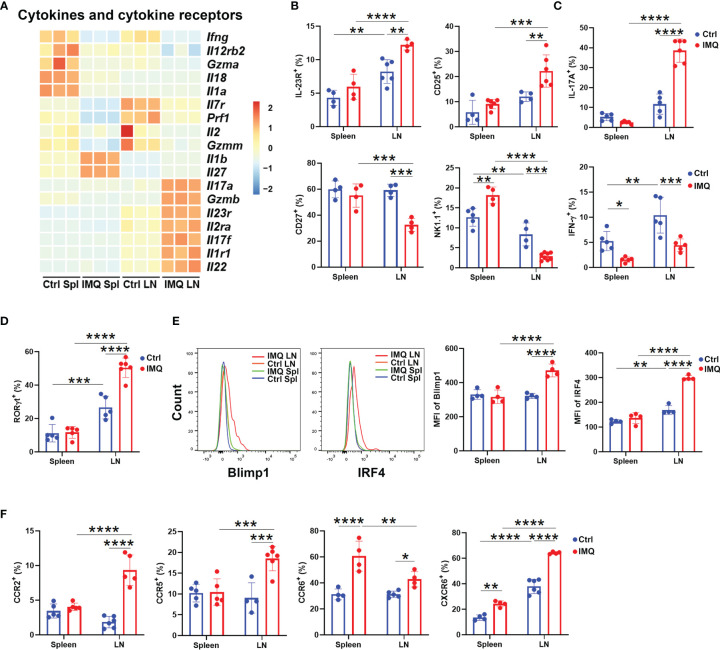
Characterization of γδ T cells in psoriatic mice. **(A)** Heatmap of cytokines and cytokine receptors of γδ T cells from different tissues. The skin-draining LN and splenic γδ T cells from healthy control and IMQ-treated mice were abbreviated as Ctrl LN, Ctrl Spl, IMQ LN, and IMQ Spl, respectively. Each sample represented γδ T cells obtained from 15 mice. **(B)** Comparison of the ratio of IL-23R^+^ (*F*=2.621), CD25^+^ (*F*=3.031), CD27^+^ (*F*=11.28) and NK1.1^+^ (*F*=39.56) populations in γδ T cells between the groups described above (N=4-6). **(C)** The expression of IL-17A (*F*=67.6) and IFN-γ (*F*=1.412) in γδ T cells was measured by flow cytometry (N=5). **(D)** The comparison of RORγt^+^ γδ T populations was performed (N=5, *F*=23.46). **(E)** Flow cytometry analysis of Blimp1 and IRF4 expression in γδ T cells obtained from the different groups described above (N=4, *F*=26.59, 55.75). **(F)** The percentages of CCR2^+^ (*F*=37.42), CCR5^+^ (*F*=11.64), CCR6^+^ (*F*=7.521), and CXCR6^+^ (*F*=19.89) populations in γδ T cells are shown (N=4-6). At least three independent experiments were performed with 4-6 mice in each group and a two-way ANOVA with Sidak’s multiple comparison was performed to compare the differences described above. *p < 0.05, **p < 0.002, ***p < 0.0002, ****p < 0.0001.

To address the inflammatory function of γδ T cells in the psoriasis model, we first investigated the cytokine and cytokine receptor signatures of γδ T cells from control and IMQ-treated mice. As shown in **Figure 3A**, the expression of cytokines from the IL-17 superfamily (*Il17a, Il17f, Il22*), cytokine receptors (*Il23r, Il2ra, Il1r1*) and *Gzmb* was exclusively higher in DLN γδ T cells from the IMQ-treated mice than in other γδ T populations (**Figure 3A**). Flow cytometry analysis also confirmed that they included more cells with positive expression of IL-23R and CD25 [molecules associated with IL-17 production (33)], but fewer cells expressing CD27 and NK1.1 (**Figure 3B** add **Figure S3A**). Indeed, upon PMA and ionomycin stimulation, the DLN γδ T cells from psoriatic mice had a significantly elevated percentage of IL-17A^+^ cells and decreased IFN-γ production (**Figure 3C** and **Figure S3B**).

We also compared the expression of transcription factors within γδ T cells of healthy and psoriatic mice. Consistent with the augmented capability of IL-17 production, RNA-seq and flow cytometry analysis both confirmed that more RORγt^+^ cells were found in γδ T cells from IMQ-treated DLNs than in γδ T cells from other groups (**Figures S4A, B** and **Figure 3D**). In addition, these pathogenic γδ T cells from the DLN of IMQ-treated mice showed higher level expression of *Prdm1, Maf, Irf4, Runx3, Stat5, and Batf*, and flow cytometry analysis further confirmed the exclusive upregulation of Blimp1 and IRF4 (**Figure S4A** and **Figure 3E**). These transcriptional data again demonstrated that the γδ T cells in DLN were imprinted by the destiny of γδ17 T cells after IMQ treatment.

Following dermatitis, both γδ T cells in the DLN and spleen upregulated the genes associated with migration (**Figure 2C**). Therefore, we also determined the expression of chemokine receptors. Notably, the transcription of *Ccr1*, *Ccr2*, *Ccr4*, *Ccr5*, *Ccr6*, and *Cxcr6* was higher in DLN γδ T cells from IMQ-treated mice than in other γδ T populations (**Figure S4A**). The increased ratios of CCR2^+^, CCR5^+^, CCR6^+^, and CXCR6^+^ cells were confirmed by flow cytometry (**Figure 3F** and **Figure S4B**). Interestingly, compared to control splenic γδ T cells, the splenic γδ T cells from IMQ-treated mice also showed increased percentages of CCR6^+^ and CXCR6^+^ cells, and their CCR6 expression was the highest between each population (**Figure 3F** and **Figure S4B**). CCR2 and CCR6 were reported to direct pathogenic γδ17 T cells to migrate into the dermis and cause inflammatory injury (18, 34). Hence, these results suggested that IMQ stimulation augmented the chemotaxis of γδ T cells from peripheral tissues to inflamed sites.

### Skin-Draining LN γδ T Cells From Psoriatic Mice Had a Higher Level of Glycolysis

**Figure 4:**
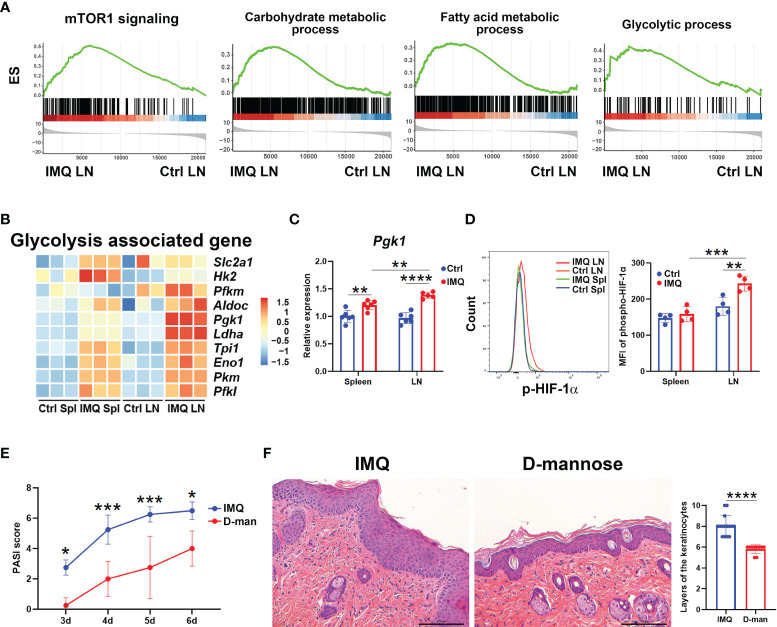
D-mannose successfully attenuated psoriatic inflammation induced by IMQ. The skin-draining LN and splenic γδ T cells from control (Ctrl) and IMQ-treated mice were abbreviated as Ctrl LN, Ctrl Spl, IMQ LN, and IMQ Spl, respectively. **(A)** Pathways significantly enriched in pathogenic DLN γδ T cells by GSEA. **(B)** Heatmap of glycolysis-associated genes in γδ T cells obtained from the groups described above. **(C)** Quantitative PCR analysis of *Pgk1* expression in γδ T cells (N=5-6, *F*=7.708). **(D)** Flow cytometry analysis of phospho-HIF-1α in γδ T cells was performed. A multiple comparison of the staining intensity of p-HIF-1α in γδ T cells is shown on the right (N=4, *F*=6.425). **(E, F)** Mice were treated with or without 20% D-mannose (D-man) given in drinking water for one week. Then, the IMQ-induced psoriatic model was established. During IMQ treatment, D-mannose was still administered orally. **(E)** Comparison of the PASI scores of psoriatic mice treated with or without D-mannose on day 3, 4, 5, and 6 after IMQ exposure (N=5, *F*=0.4766). **(F)** Representative H&E staining of skin sections obtained from psoriatic mice treated with or without D-mannose. The scale bar represents 100 μm, and the statistical analysis of the layers of keratinocytes is shown on the right (N≥10, *t*=6.985). The unpaired *t* test and two-way ANOVA with Sidak’s multiple comparison were performed to compare the differences described above. At least three independent experiments were performed with 4-6 mice in each group. *p < 0.05, **p < 0.002, ***p < 0.0002, ****p < 0.0001.

We next characterized the metabolic pattern of γδ T cells from IMQ-treated mice by gene set enrichment analysis (GSEA). After the psoriatic model was built, enrichments of genes associated with mTOR1 signaling and the metabolic processes of carbohydrates, fatty acids, glutamine family amino acids, glucose 6-phosphate, and glycolysis were observed in skin DLN γδ T cells (**Figure 4A** and **Figure S5A**). In spleen, the enriched pathways associated with metabolism were the metabolic processes for carbohydrates, glucose 6-phosphate, and cholesterol (**Figure S5A**). To meet the large energy demands for activation or differentiation, T cells usually switch their metabolic pattern to glycolysis (35, 36). Previous works have reported that the mTOR1-regulated glycolysis is required for the development and function of γδ17 T cells (35). The enrichments of pathways associated with mTOR1 signaling and glycolytic process in psoriatic DLN γδ T cells were consistent with these reports and implied the enhanced capability of glycolysis (**Figure 4A**). Hence, we determined the glycolytic signatures of γδ T cells in psoriatic mice. Upon IMQ stimulation, genes upregulated in γδ T cells tied to glycolysis included *Slc2a1*, *Hk2*, *Pfkm*, *Aldoc*, *Pgk1*, *Ldha*, *Tpi1*, *Eno1*, *Pkm*, *and Pfkl* (**Figure 4B**). Among these highly expressed genes, DLN γδ T cells from psoriatic mice exhibited the most distinctive differences in the expression of *Pgk1* and *Ldha* relative to other γδ T populations (**Figures 4B, C**). As expected, the DLN γδ T cells from psoriatic mice also expressed the highest level of p-HIF-1α, the key regulator of glycolysis (23), and our flow cytometry analysis confirmed this finding (**Figure S4A** and **Figure 4D**). Collectively, these data indicated that the skin-draining LN γδ T cells from psoriatic mice had a higher level of glycolysis.

### D-Mannose Treatment Successfully Alleviated IMQ-Induced Murine Psoriasis

D-mannose, as a C-2 epimer of glucose, has been reported to impair glycolysis and suppress the activation of HIF-1α (31); therefore, we hypothesized that it may also have a beneficial effect in the model of psoriasis. As expected, D-mannose alleviated the scaly erythematous epidermis and resulted in smaller spleens in psoriatic mice, while it didn’t affect the weight (**Figures S5B-D**). Compared to mice exposed only to IMQ, the PASI score was decreased in mannose-treated mice starting on the third day after IMQ treatment (**Figure 4E**). Histological analysis also showed that the mice given D-mannose orally had decreased inflammatory cell infiltration and hyperkeratosis (**Figure 4F**). The layers of keratinocytes in skin were decreased significantly because of D-mannose treatment (**Figure 4F**). Together, these data demonstrated that oral administration of D-mannose successfully alleviated the skin inflammation caused by IMQ exposure.

### D-Mannose Suppressed the Proliferation and IL-17 Production of γδ T Cells in Psoriatic Mice

**Figure 5:**
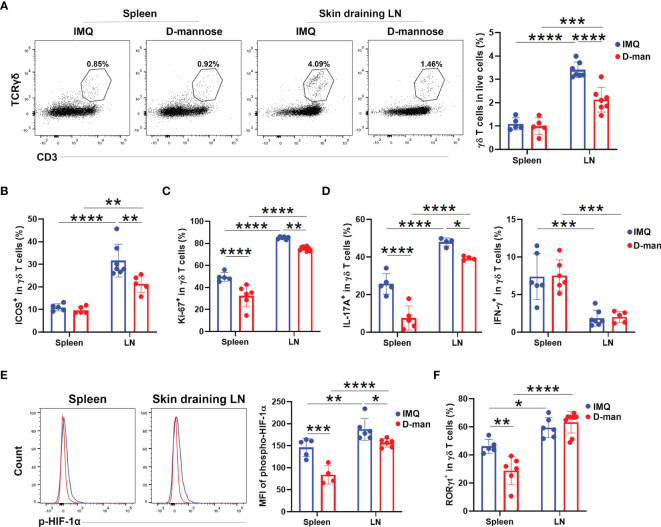
D-mannose suppressed γδ T cells *in vivo*. Mice were treated with or without 20% D-mannose (D-man) given in drinking water for one week. Then, the IMQ-induced model was built. During IMQ treatment, D-mannose was still administered orally. **(A)** Flow cytometry analysis of the percentage of γδ T cells in live cells (N=5-7). The statistical analysis is shown on the right (*F*=13.00). **(B)** Comparison of the ratio of ICOS^+^ populations in skin-draining LNs and splenic γδ T cells obtained from psoriatic mice treated with or without D-mannose (N=5-7, *F*=5.111). **(C)** Flow cytometry was used to analyze Ki-67 expression in skin-draining LNs and splenic γδ T cells obtained from the mice described above (N=5-7, *F*=3.586). **(D)** The percentages of IL-17A^+^ and IFN-γ^+^ cells in γδ T cells from skin-draining LNs and spleens were measured by flow cytometry (N=4-7, *F*=4.687). **(E)** The expression of phospho-HIF-1α in γδ T cells from different groups was determined by flow cytometry (N=4-6, *F*=3.322). **(F)** Comparison of the percentage of RORγt^+^ cells in γδ T cells obtained from the mice described above (N=5-9, *F*=11.59). The experiments were repeated at least three times and the two-way ANOVA with Sidak’s multiple comparison was used to made statistical analysis described above. *p < 0.05, **p < 0.002, ***p < 0.0002, ****p < 0.0001.

To determine whether the beneficial effect of D-mannose on psoriasis was achieved by the regulation of γδ T cells, we performed flow cytometry analysis. As shown in **Figure 5A**, mannose treatment selectively downregulated the frequency of γδ T cells in DLN from psoriatic mice. Compared to mice treated without D-mannose, mice given mannose also had a lower level of ICOS expression exclusively in DLN γδ T cells (**Figure 5B** and **Figure S6A**). Considering the decreased frequency and ICOS expression of DLN γδ T cells upon D-mannose treatment, we determined their capabilities of proliferation and cytokine production. Both spleens and DLNs from mice given mannose had reduced percentages of Ki-67^+^ and IL17A^+^ γδ T cells compared to mice treated with IMQ alone; however, the expression of IFN-γ in γδ T cells was not altered (**Figures 5C, D** and **Figure S6A**). Nevertheless, the γδ T cells from DLN contained more activated cells with positive expression of ICOS, Ki-67, and IL-17A than splenic γδ T cells after feeding with D-mannose (**Figures 5B–D** and **Figure S6A**). To examine the potential underlying machinery, we compared the transcription factors of these γδ T cells. As shown in **Figure 5E**, oral administration of D-mannose resulted in decreased expression of p-HIF-1α in the spleen and DLN compared to mice that received IMQ alone, which may imply the lowered glycolytic capabilities of γδ T cells (**Figure 5E**). However, a reduced frequency of RORγt^+^ cells was only found in the spleen of psoriatic mice treated with D-mannose (**Figure 5F** and **Figure S6A**). Together, these results suggested that D-mannose could attenuate psoriatic inflammation by suppressing the proliferation and IL-17 production of γδ T cells.

### D-Mannose Suppressed γδ T Cells by Inhibiting Glycolysis and AKT/mTOR Signaling

Previous findings by Dunfang et al. demonstrated that D-mannose could induce Treg cells and suppress autoimmune diabetes and airway inflammation (29). To verify whether the suppression of γδ T cells by D-mannose was achieved by the induction of Tregs, we examined the ratio of Treg cells in the spleen and DLN of psoriatic mice treated with or without D-mannose. Flow cytometry analysis showed that D-mannose treatment significantly increased the percentage of Treg cells in spleen; however, the ratio of Tregs in DLN was not altered (**Figure S6B**). This partially excluded the effect of Treg induction on the suppression of DLN γδ T cells by D-mannose.

**Figure 6:**
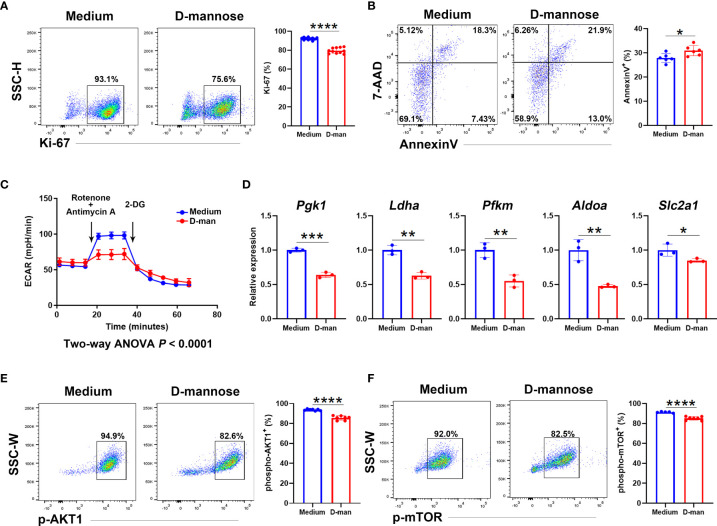
D-mannose suppressed γδ T cells by disturbing their glycolytic capabilities and AKT/mTOR signaling. **(A–F)** LN and splenic γδ T cells from at least 15 mice were pooled together and cultured *in vitro* with or without D-mannose. After three days of stimulation with anti-CD3 and anti-CD28, γδ T cells were harvested. **(A)** Flow cytometry analysis of Ki-67^+^ cells in γδ T cells was performed (N=11, *t*=12.79). **(B)** Flow cytometry was used to determine the expression of Annexin V and 7-AAD in γδ T cells (N=6, *t*=2.661). **(C)** Extracellular acidification rates (ECARs) were measured to determine the glycolytic capacity in γδ T cells. **(D)** Quantitative PCR analysis of glycolytic genes expression in γδ T cells cultured with or without D-mannose (N=3). **(E, F)** Flow cytometry was used to analyze the expression of phospho-AKT1 (*t*=9.252) and phospho-mTOR (*t*=7.152) in γδ T cells cultured with or without D-mannose (N=5-7). The experiments described above were repeated at least three times and the unpaired *t* test was used to compare the differences described above. *p < 0.05, **p < 0.002, ***p < 0.0002, ****p < 0.0001.

We next investigated the direct effect of D-mannose on γδ T cells. γδ T cells sorted from spleens and LNs were pooled together and cultured *in vitro*. After three days of TCR stimulation, γδ T cells treated with D-mannose contained fewer cells with positive expression of Ki-67 and more apoptotic cells (**Figures 6A, B**). To reveal the underlying mechanism, we hypothesized that the suppression of γδ T cells may result from alterations in metabolism and performed a glycolytic rate assay. Indeed, the lower extracellular acidification rate (ECAR) indicated that D-mannose significantly suppressed the glycolytic capabilities of γδ T cells, while quantitative PCR showed that D-mannose reduced the expression of key glycolytic molecules in γδ T cells, such as *Pgk1*, *Ldha*, *Pfkm*, *Aldoa* and *Slc2a1* (**Figures 6C, D**). Moreover, both flow cytometry analysis and capillary western blot illustrated the expression of phospho-AKT and phospho-mTOR was downregulated in γδ T cells after culture with D-mannose (**Figures 6E, F** and **Figure S7**). Together, considering the crucial roles of AKT/mTOR signaling in glycolysis (35, 37), our results suggested that D-mannose treatment contributed to the lower proliferation and higher apoptosis of γδ T cells by impairing glycolysis *via* the AKT/mTOR axis.

## Discussion

Skin is the crucial barrier that protects us from drastic changes in the external environment (38). Among the immunological populations of skin, γδ T cells have received more attentions in recent years (13). Here, we demonstrated that the skin DLN γδ T cells from psoriatic mice became highly activated/effector γδ T cells with the phenotype of γδ17 T cells. Most importantly, we determined that D-mannose, a hexose sugar, could alleviate the experimental psoriasis by suppressing γδ T cells *via* inhibition of glycolysis and AKT/mTOR/HIF-1α signaling.

Emerging evidence has shown that inflammatory γδ17 T cells migrating between the DLN and dermis play critical roles in the pathologies of psoriasis (15, 18). In accordance with previous reports (16, 18), our study found that the γδ T cells from DLN of IMQ-treated mice had augmented proliferation compared with healthy controls. Meanwhile, these activated γδ T cells acquired higher levels of ICOS, PD1, IL-23R, CD25, and IL-17A expression [phenotypes correlated to γδ17 T cells (33)], but lower levels of CD27, NK1.1, and IFN-γ expression [phenotypes correlated to IFN-γ-producing γδ T cells (33)]. In addition, the transcription factors *Irf4*, *Maf*, *Runx3*, and *Rorc*, which control the commitment of Th17 cells, were selectively elevated in γδ T cells from the DLN, upon IMQ stimulation. In murine psoriasis, γδ T cells migrate into lesions *via* CCR2 and CCR6; moreover, they can travel to distal noninflamed skin and LNs to provide memory activation under restimulation (18, 34, 39). In addition to the higher expression of CCR2 and CCR6, γδ T cells from the DLN of psoriatic mice also contained more cells with positive expression of CCR5 and CXCR6, which suggested that the inflammatory γδ T cells from the skin DLN may travel to other organs, such as the liver, gastrointestine, synovial joints, and neural system, to disturb the local immune microenvironment (40–42). The aberrant chemotaxis should be a pivotal factor that linked psoriasis to other sequelae (such as asthma and psoriatic arthritis) (43, 44). Moreover, we also found that the splenic γδ T cells of psoriatic mice had the highest level of CCR6 expression, which implied that they were motivated to migrate into the inflamed skin or DLNs. Thus, our study characterized γδ T cells in IMQ-treated mice and again underlined the accumulation of γδ17 T cells in the skin draining LN of a psoriatic model.

Metabolic reprogramming dictates the fate of T cells, and interference with the metabolic state of T cells could orchestrate the immune reaction (21, 36, 45). Compared to other γδ T populations, γδ T cells from the DLN of psoriatic mice acquired the highest p-HIF-1α expression and upregulated genes associated with glycolysis and mTOR1 signaling. HIF-1α-dependent glycolysis is implicated in the pathogenesis of Th17 cells; hence, suppressing the activation of HIF-1α and impeding the correlated glycolysis may offer protection in the mouse model of psoriasis (25, 37).

In recent years, D-mannose was reported to suppress immunopathology in models such as lupus, diabetes and colitis (29–31). Treatment with D-mannose effectively impaired succinate-mediated HIF-1α activation and glucose metabolism in macrophages (31). As in T cells, the addition of mannose *in vitro* also resulted in a decreased capacity to utilize glycolysis, and induced the differentiation of Treg cells (29). However, the effects of D-mannose in psoriasis and γδ T cells have not been elucidated. Our study first showed that D-mannose could alleviate the pathology of IMQ-induced psoriasis by suppressing proliferation and IL-17 production in γδ T cells. In particular, D-mannose selectively reduced the frequency of γδ T cells in skin DLN. Although the percentage of Treg cells was increased in spleen, the ratio of Tregs in DLN was not altered by D-mannose treatment. This partially excluded the suppression of DLN γδ T cells was achieved by the induction of Treg cells.

Further *in vitro* culture indicated that the addition of D-mannose disturbed glycolysis and decreased the expression of p-AKT and p-mTOR in γδ T cells. AKT/mTOR signaling participates in the progression of psoriasis and tunes the fate of T cell by HIF-1α-mediated glycolysis (46–48). mTOR1 is required for the proliferation and survival of peripheral Vγ4^+^ γδ T cells, while mTOR1 and mTOR2 potentiate γδ17 T cells by regulating glycolysis and mitochondrial ROS production (35). Hence, the prevention of glycolysis *via* downregulation of AKT/mTOR signaling may explain the suppression of γδ T cells’ proliferation and survival by D-mannose. In accordance with these results, D-mannose treatment also resulted in decreased expression of the glycolytic regulator p-HIF-1α in γδ T cells from IMQ-treated mice. This again implied the crucial role of impeding AKT/mTOR/HIF-1α-mediated glycolysis in the suppression of psoriatic γδ T cells by D-mannose. ICOS, as a costimulatory molecule, could activate mTOR signaling to promote follicular helper T cell response *via* driving glycolysis, lipogenesis, and Glut1-mediated glucose metabolism (49). In our findings, γδ T cells from the DLN of psoriatic mice included more cells with positive expression of ICOS. Interestingly, similar to the alterations in γδ T cells frequency, oral D-mannose specifically decreased the expression of ICOS in γδ T cells from the DLN rather than the spleen. Considering ICOS could promote the activation of mTOR signaling (49), it is thus likely that ICOS may transfer upstream signaling in the process by which D-mannose regulates γδ T cells. Together, our results suggested that D-mannose suppressed psoriatic pathogenic γδ T cells by interfering with AKT/mTOR/HIF-1α signaling-mediated glycolysis.

Psoriasis is a chronic disease with intricate mechanisms (6). Although we demonstrated that D-mannose alleviated psoriasis by interfering with glycolysis in γδ T cells, there are still limitations. The psoriatic skin consists of overproliferative keratinocytes that can cross-talk with T cells to exacerbate the pathology. Previous works have reported that these keratinocytes also have higher glucose metabolism and inhibition of glycolysis can alleviate IMQ-induced psoriasis by inhibiting their proliferation (50). In our work, we cannot exclude the possibility that the remission of psoriasis achieved by D-mannose treatment partially resulted from the suppression of glycolysis in pathogenic cells such as keratinocytes. Hence, the mechanisms underlying the alleviation of psoriasis by D-mannose still need further exploration, and other target cells of D-mannose should also be verified in the future.

Overall, our study explicitly characterized the phenotype and function of γδ T cells in IMQ-treated mice. We found the skin-draining LN γδ T cells were activated, and imprinted by the destiny of γδ17 T cells during psoriasis. These pathogenic γδ T populations attained highly p-HIF-1α expression, while upregulated pathways associated with glycolysis and mTOR1 signaling. Most importantly, we discovered that D-mannose, a previously reported inhibitor of HIF-1α and glucose metabolism, had a beneficial effect on psoriasis by restraining the pathogenic γδ17 T cells. The impediment of AKT/mTOR/HIF-1α-mediated glycolysis may contribute to the suppression achieved by D-mannose. These findings supply a new method for manipulation of γδ T cells, and will further warrant the exploration of D-mannose in the clinical application of skin immunological disorders.

## Data Availability Statement

The datasets presented in this study can be found in online repositories. The names of the repository/repositories and accession number(s) can be found below: https://www.ncbi.nlm.nih.gov/geo/, GSE188905.

## Ethics Statement

The animal study was reviewed and approved by Animal Ethics Committee of Beijing Friendship Hospital, Capital Medical University.

## Author Contributions

ML, HC, and DT participated in performing the research, analyzing the data, and initiating the original draft of the article. LY, XD, and YP participated in performing the research. XM and DZ established the hypotheses, supervised the studies, analyzed the data, and co-wrote the manuscript. All listed authors participated meaningfully in the study and that they have seen and approved the submission of this manuscript.

## Funding

Grants from the National Natural Science Foundation of China (No. 81870399 and 82100670), Youth Beijing Scholar (No. 035), and Beijing Nova Program (Z211100002121036) supported this work.

## Conflict of Interest

The authors declare that the research was conducted in the absence of any commercial or financial relationships that could be construed as a potential conflict of interest.

## Publisher’s Note

All claims expressed in this article are solely those of the authors and do not necessarily represent those of their affiliated organizations, or those of the publisher, the editors and the reviewers. Any product that may be evaluated in this article, or claim that may be made by its manufacturer, is not guaranteed or endorsed by the publisher.

## Supplementary Material

Supplementary Figure 1 Gating strategy of γδ T cells. The dot plots represented the gating strategy of γδ T cells in LN, which was also used to gate the splenic γδ T cells. After find out the live single lymphocytes, γδ T cells (TCRγδ^+^) were gated in CD3^+^ cells.

Supplementary Figure 2 Comparison of skin-draining LNs and splenic γδ T cells in healthy and psoriatic mice. **(A)** Dendrogram of γδ T cells obtained from spleen and skin-draining LNs in control (Ctrl) and IMQ-treated mice. **(B**) Pathways significantly enriched in IMQ LN vs. IMQ Spl by GO analysis. **(C)** Flow cytometry analysis of Ki-67, CD44, CD62L, ICOS, and PD1 expression in γδ T cells from the groups described above (N=4-6). The experiments were repeated at least three times with 4-6 mice in each group.

Supplementary Figure 3 Skin-draining LN γδ T cells from psoriatic mice showed the phenotype of γδ17 T cells. **(A)** Flow cytometry analysis of IL-23R, CD25, NK1.1, and CD27 expression in γδ T cells from spleen (Spl) and skin draining LN of control (Ctrl) or IMQ-treated mice (N=4-6). **(B)** Flow cytometry analysis of IL-17A and IFN-γ in γδ T cells from the groups described above after PMA and ionomycin stimulation (N=5). The experiments described above were repeated three times.

Supplementary Figure 4 Comparison of transcription factors, chemokines and chemokine receptors in skin-draining LN and splenic (Spl) γδ T cells from control (Ctrl) or IMQ-treated mice. **(A)** Heatmap of transcription factors, chemokines and chemokine receptors in γδ T cells from different groups. The splenic and skin-draining LN γδ T cells from control or IMQ-treated mice were shortened to Ctrl Spl, Ctrl LN, IMQ Spl and IMQ LN, respectively. **(B)** Flow cytometry analysis of RORγt, CCR2, CCR5, CCR6 and CXCR6 expression in γδ T cells from the groups described above (N=4-6). The experiments were repeated three times with at least 4–6 mice in each group.

Supplementary Figure 5 D-mannose had a beneficial effect on IMQ-induced psoriasis. **(A)** GSEA of pathways associated with metabolism. The γδ T cells of spleen and skin draining LN from control (Ctrl) or IMQ-treated mice were shortened as Ctrl Spl, Ctrl LN, IMQ Spl and IMQ LN, respectively. **(B-D)** Mice were orally given drinking water with or without 20% D-mannose for one week. Then, the IMQ-induced model was established, while D-mannose was still administered orally. After 6 consecutive days of IMQ treatment, the mice were harvested. Pictures of mice **(B)** given drinking water with or without D-mannose and the spleens of these mice **(C)** are shown. The comparison of weight in psoriatic mice treated with or without D-mannose was performed (N=5) **(D)**. At least three independent experiments were repeated.

Supplementary Figure 6 Impact of D-mannose on γδ T cells obtained from psoriatic mice. **(A)** Flow cytometry analysis of ICOS, Ki-67, IL-17A, IFN-γ, and RORγt expression in splenic and skin-draining LN γδ T cells from psoriatic mice given with or without D-mannose (N=4-9). **(B)** Flow cytometry was used to determine CD25 and Foxp3 expression in CD4^+^ T cells (N=4-5). The multiple comparison (two-way ANOVA) of the ratio of CD25^+^Foxp3^+^ cells in CD4^+^ T cells is shown on the right (*F*=6.235). At least three independent experiments were performed with 4-9 mice in each group.

Supplementary Figure 7 The capillary western blot analysis of p-AKT, p-mTOR, and β-acitn expression in γδ T cells cultured with or without D-mannose. γδ T cells obtained from spleens and LNs were pooled together and cultured with or without D-mannose. Each sample represented γδ T cells obtained from 5 mice. After 3 days of culture, the expressions of p-AKT, p-mTOR, and β-acitn in γδ T cells were evaluated using capillary western blot.
