# Supplementary figures and images for: D-Mannose Suppresses γδ T Cells and Alleviates Murine Psoriasis
Source: Front Immunol. 2022 Feb 28;13:840755. doi: 10.3389/fimmu.2022.840755 (PMC8918796; doi:10.3389/fimmu.2022.840755)

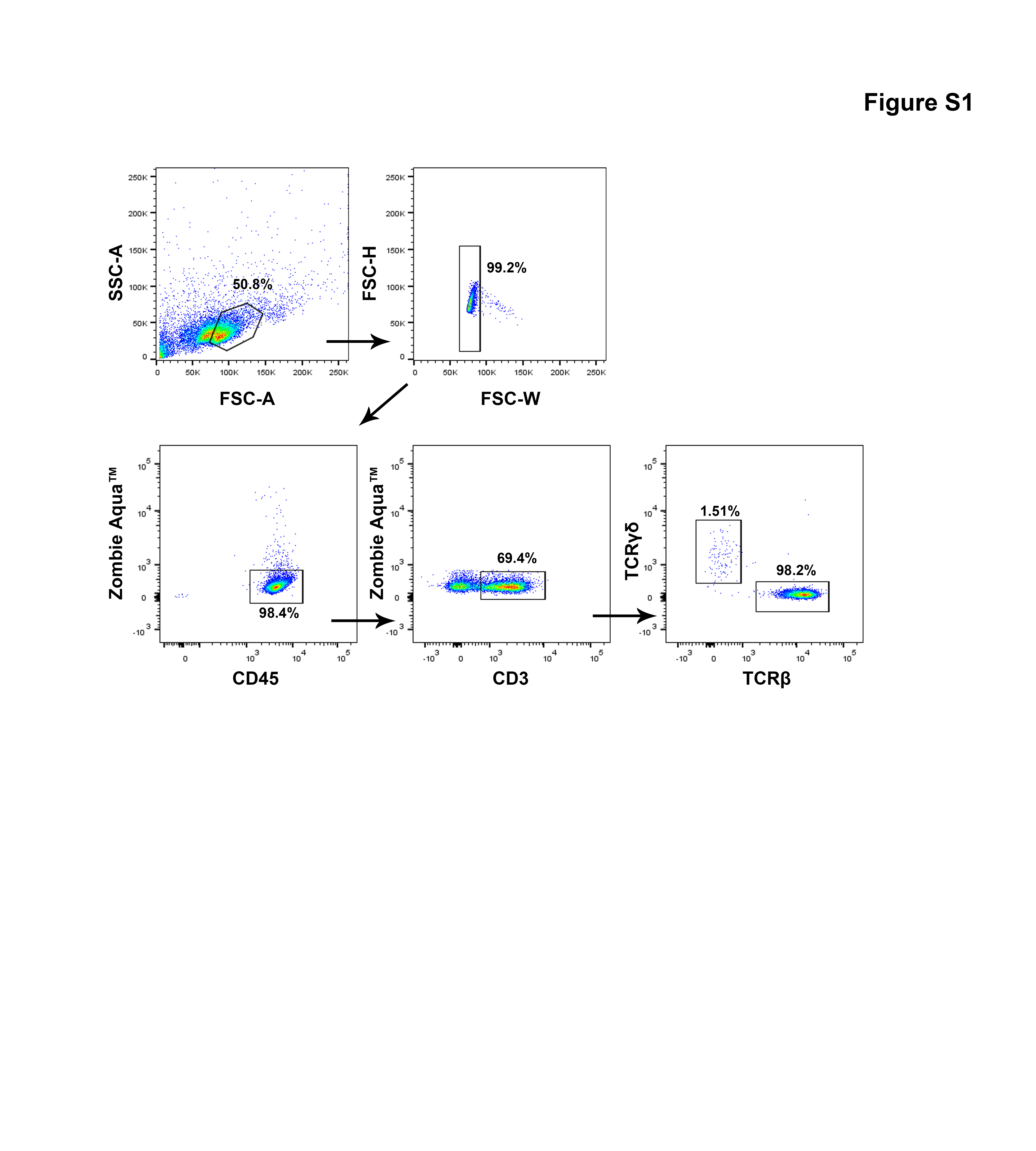

Supplement: Supplementary Figure 1 — Gating strategy of γδ T cells. The dot plots represented the gating strategy of γδ T cells in LN, which was also used to gate the splenic γδ T cells. After find out the live single lymphocytes, γδ T cells (TCRγδ+) were gated in CD3+ cells. [file Image_1.tif]

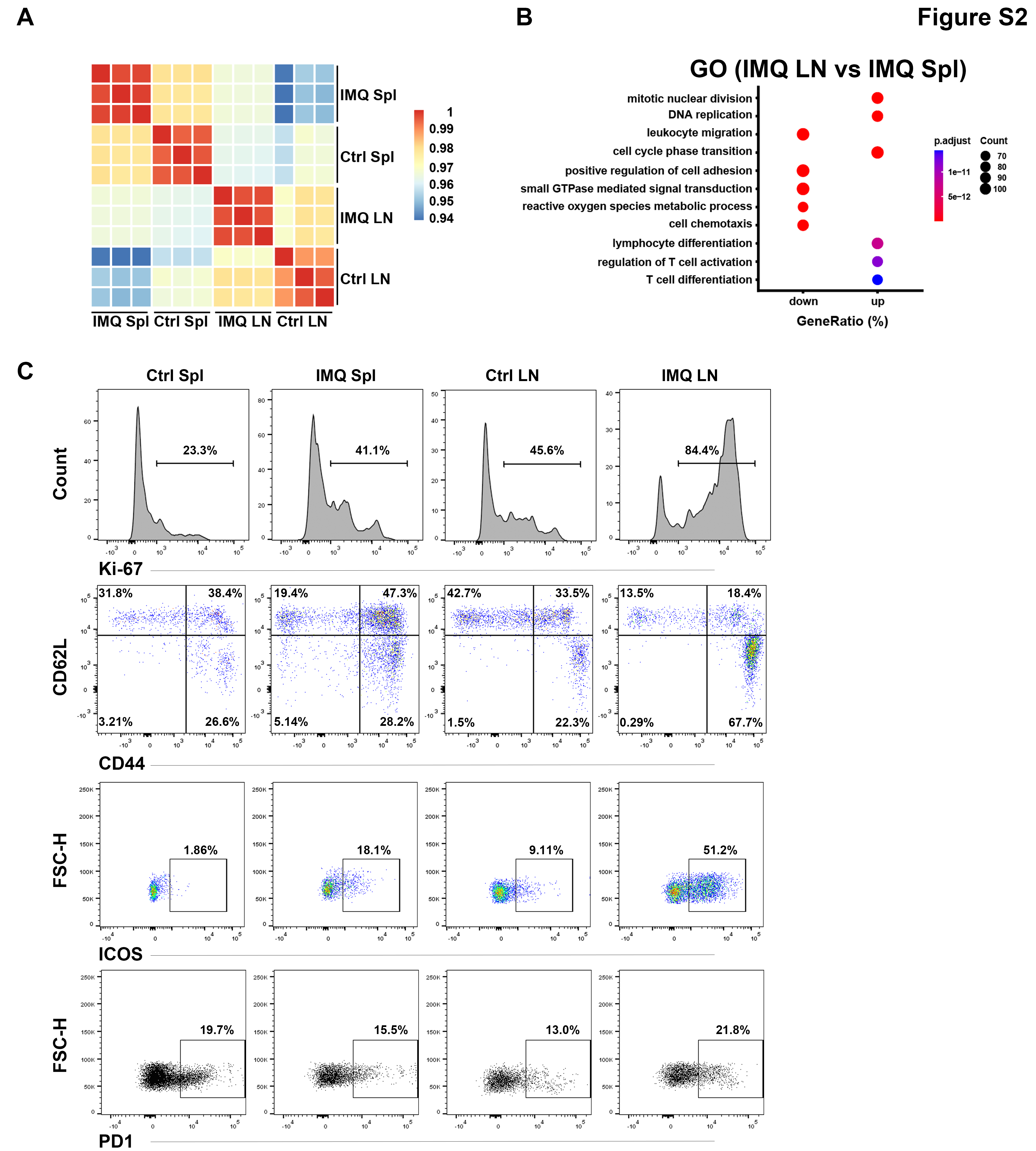

Supplement: Supplementary Figure 2 — Comparison of skin-draining LNs and splenic γδ T cells in healthy and psoriatic mice. (A) Dendrogram of γδ T cells obtained from spleen and skin-draining LNs in control (Ctrl) and IMQ-treated mice. (B) Pathways significantly enriched in IMQ LN vs. IMQ Spl by GO analysis. (C) Flow cytometry analysis of Ki-67, CD44, CD62L, ICOS, and PD1 expression in γδ T cells from the groups described above (N=4-6). The experiments were repeated at least three times with 4-6 mice in each group. [file Image_2.tif]

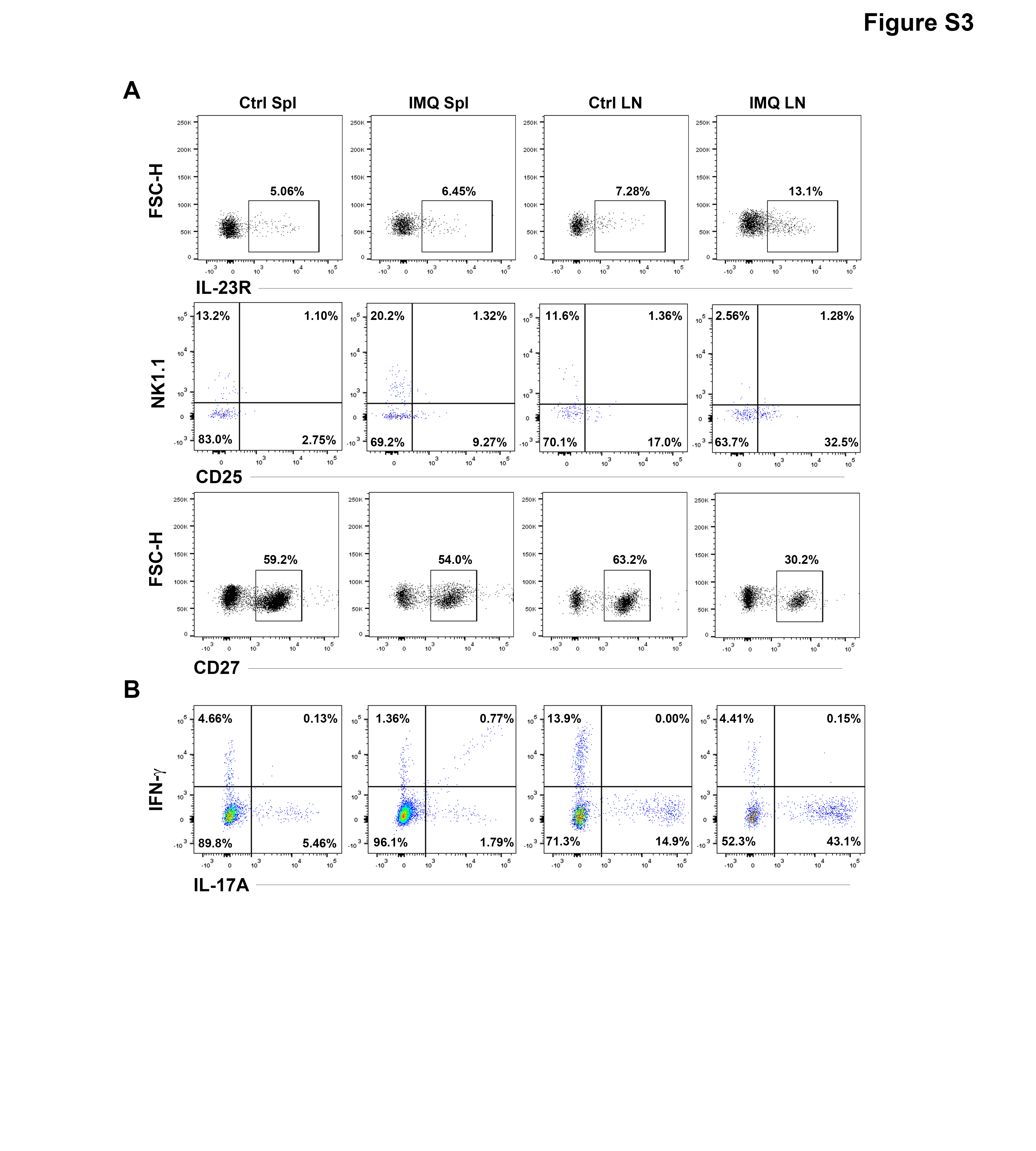

Supplement: Supplementary Figure 3 — Skin-draining LN γδ T cells from psoriatic mice showed the phenotype of γδ17 T cells. (A) Flow cytometry analysis of IL-23R, CD25, NK1.1, and CD27 expression in γδ T cells from spleen (Spl) and skin draining LN of control (Ctrl) or IMQ-treated mice (N=4-6). (B) Flow cytometry analysis of IL-17A and IFN-γ in γδ T cells from the groups described above after PMA and ionomycin stimulation (N=5). The experiments described above were repeated three times. [file Image_3.tif]

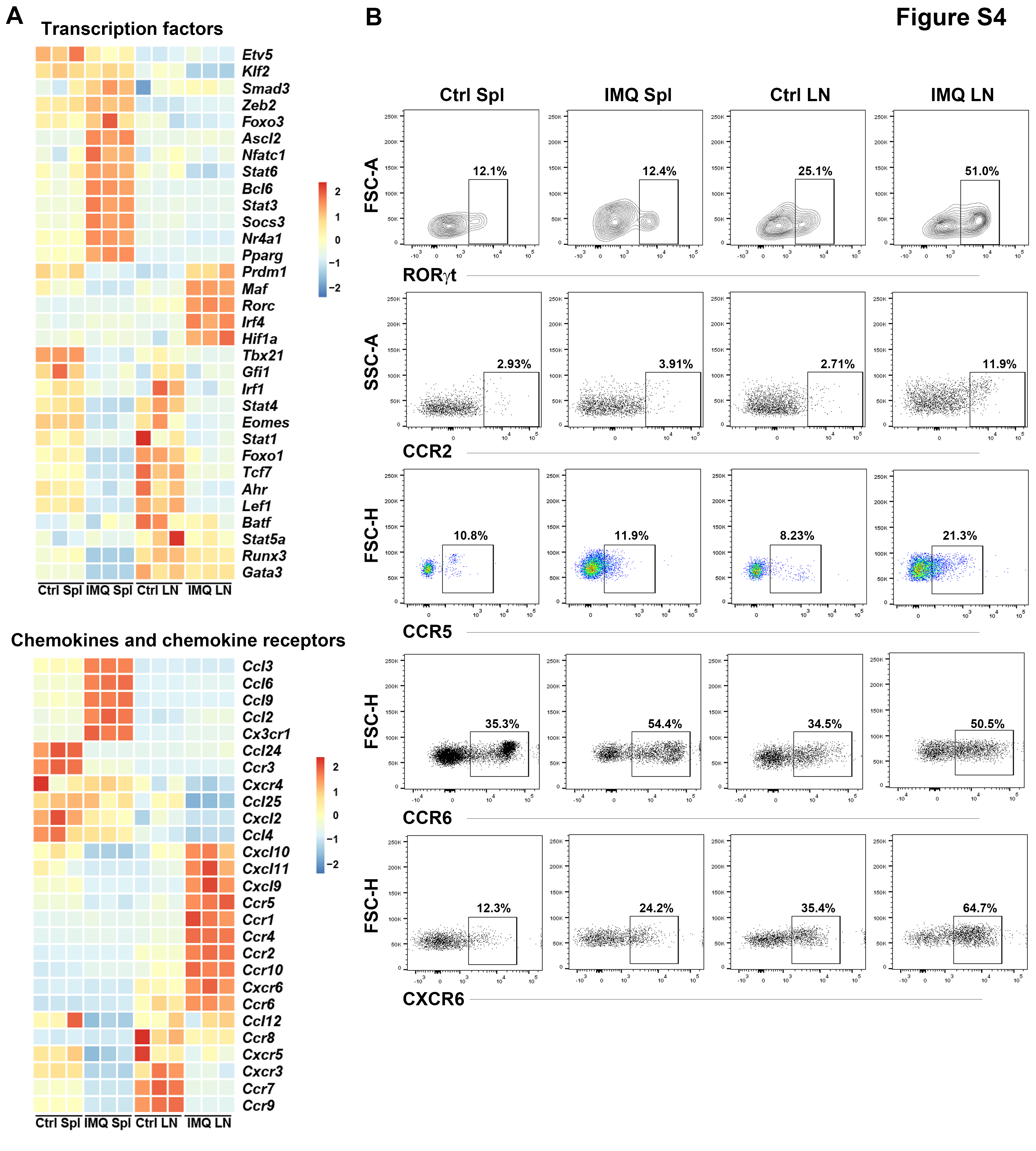

Supplement: Supplementary Figure 4 — Comparison of transcription factors, chemokines and chemokine receptors in skin-draining LN and splenic (Spl) γδ T cells from control (Ctrl) or IMQ-treated mice. (A) Heatmap of transcription factors, chemokines and chemokine receptors in γδ T cells from different groups. The splenic and skin-draining LN γδ T cells from control or IMQ-treated mice were shortened to Ctrl Spl, Ctrl LN, IMQ Spl and IMQ LN, respectively. (B) Flow cytometry analysis of RORγt, CCR2, CCR5, CCR6 and CXCR6 expression in γδ T cells from the groups described above (N=4-6). The experiments were repeated three times with at least 4–6 mice in each group. [file Image_4.tif]

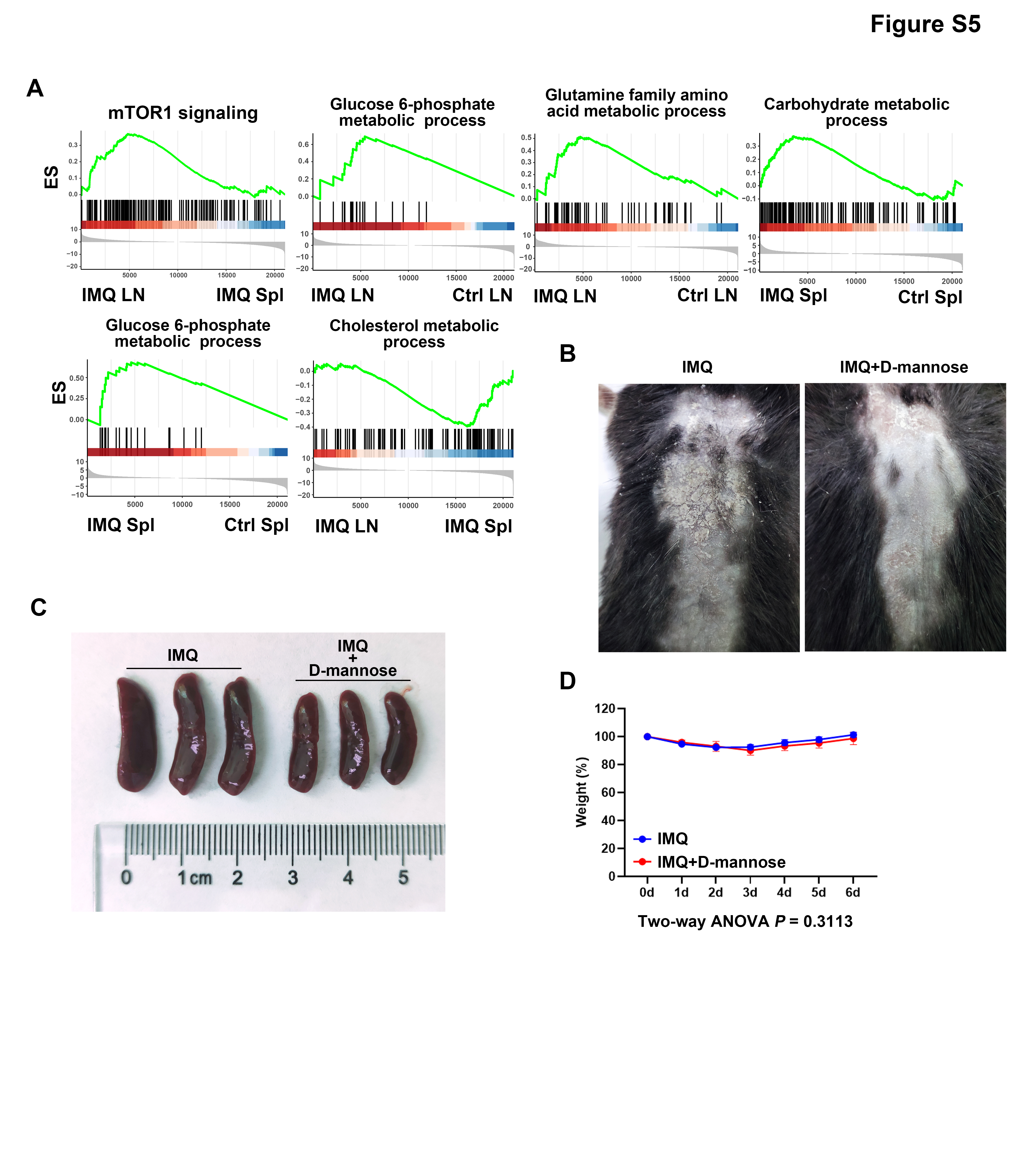

Supplement: Supplementary Figure 5 — D-mannose had a beneficial effect on IMQ-induced psoriasis. (A) GSEA of pathways associated with metabolism. The γδ T cells of spleen and skin draining LN from control (Ctrl) or IMQ-treated mice were shortened as Ctrl Spl, Ctrl LN, IMQ Spl and IMQ LN, respectively. (B-D) Mice were orally given drinking water with or without 20% D-mannose for one week. Then, the IMQ-induced model was established, while D-mannose was still administered orally. After 6 consecutive days of IMQ treatment, the mice were harvested. Pictures of mice (B) given drinking water with or without D-mannose and the spleens of these mice (C) are shown. The comparison of weight in psoriatic mice treated with or without D-mannose was performed (N=5) (D). At least three independent experiments were repeated. [file Image_5.jpeg]

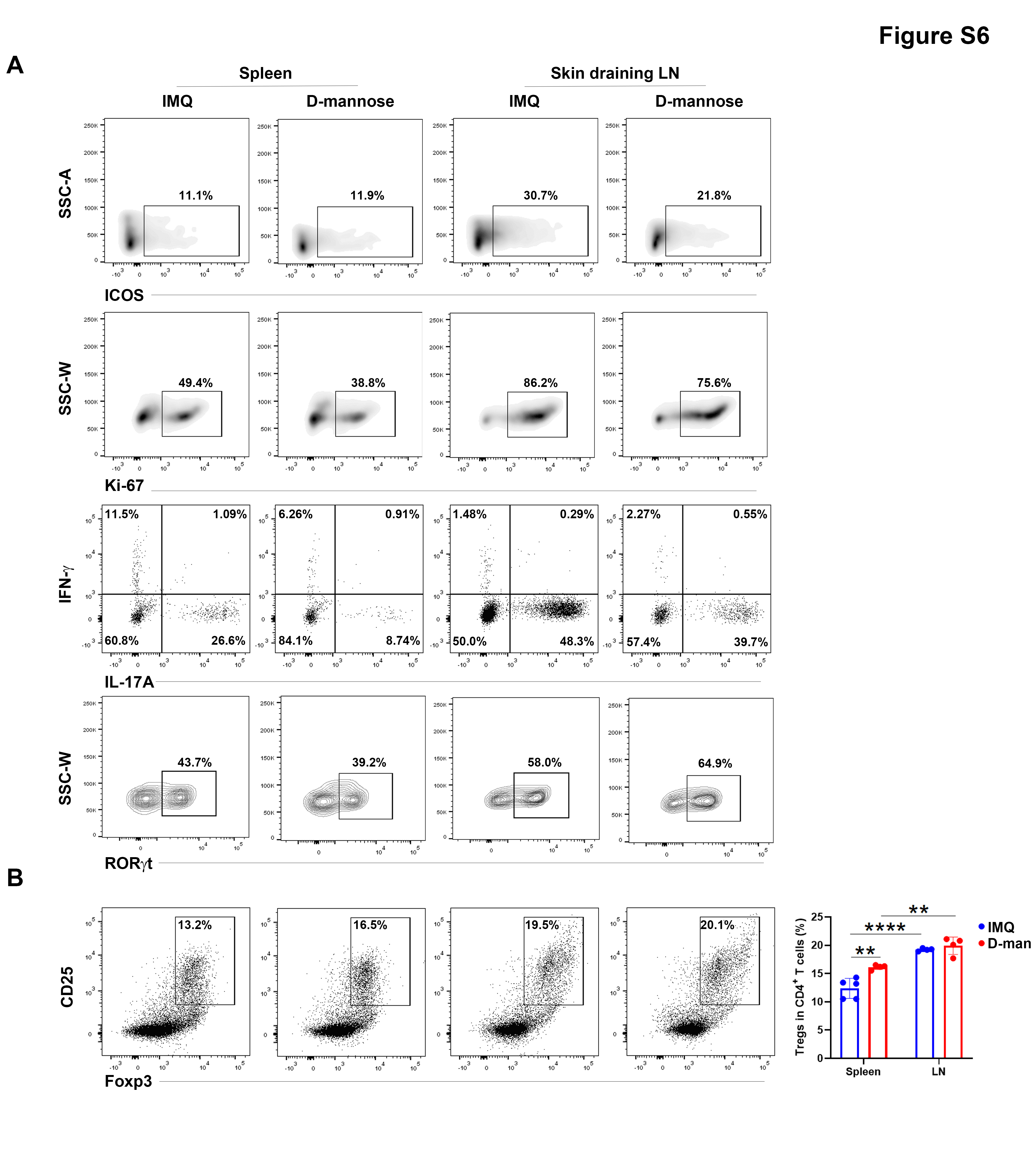

Supplement: Supplementary Figure 6 — Impact of D-mannose on γδ T cells obtained from psoriatic mice. (A) Flow cytometry analysis of ICOS, Ki-67, IL-17A, IFN-γ, and RORγt expression in splenic and skin-draining LN γδ T cells from psoriatic mice given with or without D-mannose (N=4-9). (B) Flow cytometry was used to determine CD25 and Foxp3 expression in CD4+ T cells (N=4-5). The multiple comparison (two-way ANOVA) of the ratio of CD25+Foxp3+ cells in CD4+ T cells is shown on the right (F=6.235). At least three independent experiments were performed with 4-9 mice in each group. [file Image_6.tif]

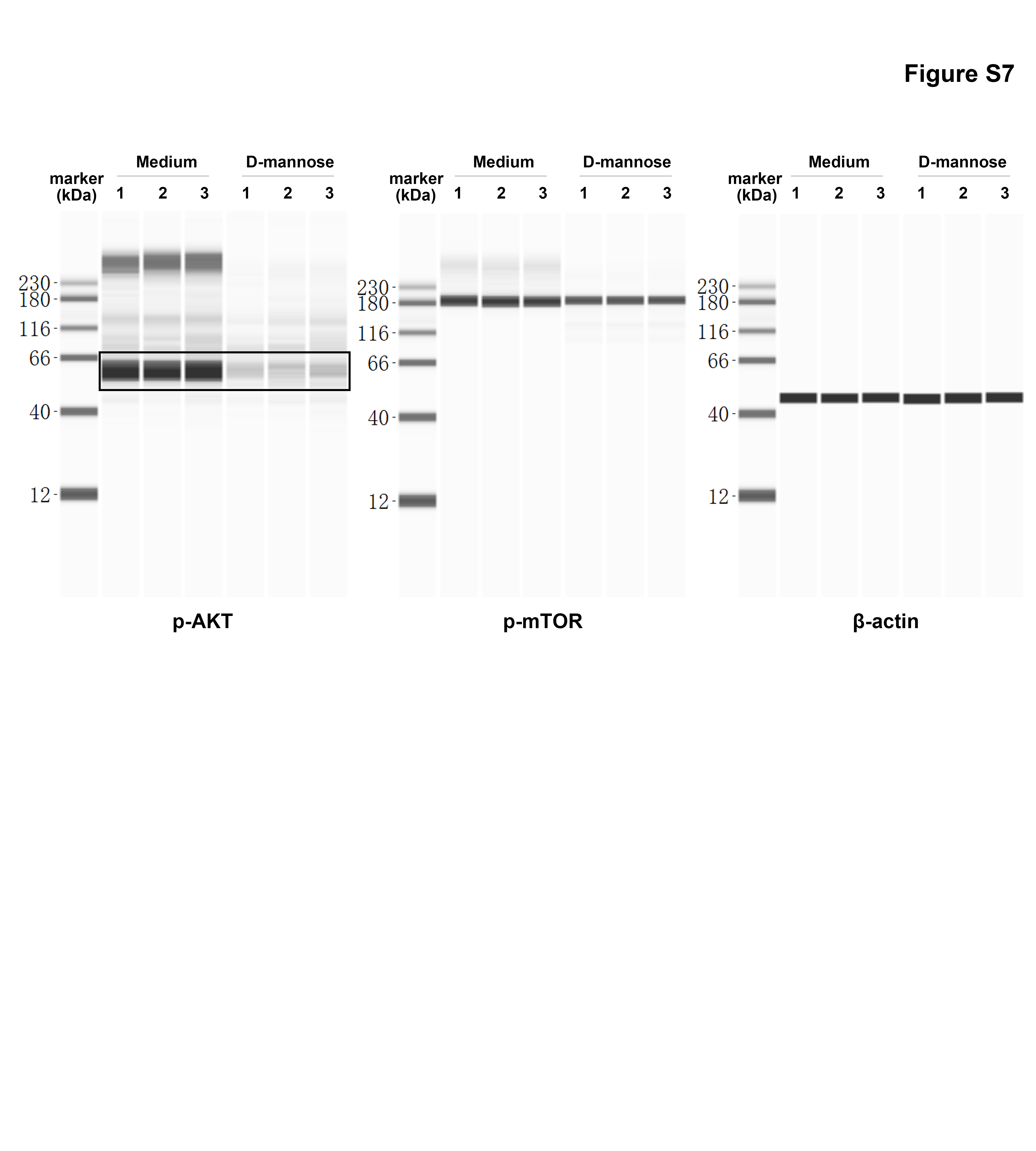

Supplement: Supplementary Figure 7 — The capillary western blot analysis of p-AKT, p-mTOR, and β-acitn expression in γδ T cells cultured with or without D-mannose. γδ T cells obtained from spleens and LNs were pooled together and cultured with or without D-mannose. Each sample represented γδ T cells obtained from 5 mice. After 3 days of culture, the expressions of p-AKT, p-mTOR, and β-acitn in γδ T cells were evaluated using capillary western blot. [file Image_7.tif]
