# Supplementary material for: D-Mannose Suppresses γδ T Cells and Alleviates Murine Psoriasis
Source: Front Immunol. 2022 Feb 28;13:840755. doi: 10.3389/fimmu.2022.840755 (PMC8918796; doi:10.3389/fimmu.2022.840755)
Supplement: Supplementary file 8 [file Table_1.docx]

**Supplemental Tables**

**Table S1. Primers for qPCR analyses**

| Primer name | Primer sequence (5’ to 3’) |
| --- | --- |
| *Ldha-*F | AGGCGGCTACACGTACAC |
| *Ldha*-R | AGCACCAACCCCAACAACTG |
| *Pgk1*-F | CCCTTCCTGGCTATCTTGGG |
| *Pgk1*-R | GGCCAGTCTTGGCATTCTCA |
| *Pfkm*-F | ACAGTGGCCGTGATGAATGT |
| *Pfkm*-R | AGCCCCCAATGATGACAAGG |
| *Aldoa*-F | GGGGTCACTTTCCTGTCTGG |
| *Aldoa*-R | TTCTCCTTCTTCCCACCCCA |
| *Slc2a1*-F | ATGGATCCCAGCAGCAAGAAG |
| *Slc2a1*-R | AGCGGTGGTTCCATGTTTGA |
| *β-actin*-F | CGATATCGCTGCGCTGGTC |
| *β-actin*-R | AGGTGTGGTGCCAGATCTTC |
